# Process Intensification for an Insect Antimicrobial Peptide Elastin-Like Polypeptide Fusion Produced in Redox-Engineered *Escherichia coli*

**DOI:** 10.3389/fbioe.2019.00150

**Published:** 2019-06-27

**Authors:** Mathias Joachim, Nicolas Maguire, Johannes Schäfer, Doreen Gerlach, Peter Czermak

**Affiliations:** ^1^Department of Life Science Engineering, Institute of Bioprocess Engineering and Pharmaceutical Technology, University of Applied Sciences Mittelhessen, Giessen, Germany; ^2^Faculty of Biology and Chemistry, Justus Liebig University, Giessen, Germany; ^3^Department of Bioresources of Fraunhofer, Institute for Molecular Biology and Applied Ecology IME, Giessen, Germany

**Keywords:** chemically defined minimal medium, disulfide bonds, rosetta gami, glutathione reductase, thioredoxin reductase, insect metalloproteinase inhibitor (IMPI)

## Abstract

Peptides and proteins containing disulfide bonds can be produced in *Escherichia coli* by targeting the oxidizing periplasm, co-expressing isomerases or chaperons, refolding from inclusion bodies, or by using redox-engineered *E. coli* strains. Thus far, protein expression in glutathione reductase and thioredoxin reductase deficient (Δ*gor* Δ*trxB*) *E. coli* strains has required a complex medium. However, a chemically defined medium suitable for large-scale production would be preferable for industrial applications. Recently, we developed a minimal medium supplemented with iron (M9i) for high-density cultivation using *E. coli* Rosetta gami B(DE3)pLysS cells. Here we show that M9i is suitable for the production of insect metalloproteinase inhibitor (IMPI), which contains five disulfide bonds, in the same *E. coli* strain. We demonstrated the scalability of the new fed-batch process by combining the scale-up criteria of constant dissolved oxygen (DO) and matching volumetric power inputs (*P/V*) at the borders of the stirrer cascade. Process intensification was achieved by investigating production feed rates and different induction times. We improved product titers by ~200-fold compared to the standard process in complex medium while maintaining the activity of the IMPI protein. Our results show for the first time that it is possible to produce active proteins containing multiple disulfide bonds in a Δ*gor* Δ*trxB E. coli* strain using M9i medium. The success of scale-up and process intensification shows that the industrial production of complex recombinant proteins in such strains using chemically defined M9i minimal medium is feasible.

## Introduction

The spread of antibiotic-resistant pathogens due to the overuse of conventional antibiotics is a severe threat to healthcare systems worldwide (Goossens et al., 2005; Costelloe et al., 2010; Klein et al., 2018). Antimicrobial peptides (AMPs) have been proposed as a potential solution to the demand for new antibiotics (Joerger, 2003; Hancock and Sahl, 2006; Pöppel et al., 2015; Bolouri Moghaddam et al., 2016) and insects produce the broadest spectrum of AMPs, therefore offering a valuable resource for the isolation of peptides with medical applications (Mylonakis et al., 2016; Tonk and Vilcinskas, 2016). Most pharmaceutical proteins are produced in recombinant microbes or eukaryotic cell lines (Sanchez-Garcia et al., 2016), and AMPs have therefore been produced in *Escherichia coli* (Hoffmann et al., 2019) as well as insect cells (Zitzmann et al., 2018).

Proteins with multiple disulfide bonds can be difficult to produce in bacteria, but AMPs with disulfide bonds have been produced in *E. coli* strains engineered with an oxidizing cytoplasm (Berkmen, 2012; Zhang et al., 2014), by co-expression with disulfide bond isomerases (Gaciarz et al., 2017) or as inclusion bodies (Hoffmann et al., 2019). Although inclusion bodies reduce the toxicity of AMPs toward host bacteria, the refolding procedure is labor-intensive and requires extensive optimization. In many cases, only 15–25% of the inclusion body material can be refolded into active proteins (Singh and Panda, 2005). Recombinant proteins with disulfide bonds can be targeted to the periplasm, or to the cytoplasm of redox-engineered *E. coli* cells. However, the periplasm makes up to only 8–16% of the total cell volume, whereas the cytoplasm accounts for most of the cell volume and contains more than 30% of the total cellular protein, making it a preferable accumulation site.

Thus far, redox-engineered *E. coli* strains for the production of recombinant proteins have required complex media containing ingredients such as yeast extract and peptone, which makes them unsuitable for large-scale production in industry (Gaciarz et al., 2017). We previously described an iron-enriched chemically defined minimal medium (M9i) which supports the cultivation of *E. coli* at high cell densities (Joachim et al., 2018). Here we tested the ability of M9i medium to support the growth of a glutathione reductase and thioredoxin reductase deficient (Δ*gor* Δ*trxB*) *E. coli* strain for the cytoplasmic production of recombinant proteins with disulfide bonds. As a model product, we expressed the insect metalloprotease inhibitor (IMPI), an AMP with five disulfide bonds (Wedde et al., 1998), fused to an elastin-like polypeptide (ELP) to facilitate product recovery. ELPs contain a repetitive pentapeptide (VPGXG), where the guest residue X can be any amino acid except proline, which allows reversible precipitation at a given transition temperature (Meyer and Chilkoti, 1999; Trabbic-Carlson et al., 2004; Banki et al., 2005; Hassouneh et al., 2010). We created a fed-batch process with a stirrer and aeration cascade for dissolved oxygen (DO) control. We then scaled up the process using the combined transfer criteria of constant DO and matching volumetric power inputs at the borders of the stirrer cascade. Using this approach, we investigated the industrial potential of redox-engineered *E. coli* combined with iron-enriched chemically defined M9i minimal medium at different feed rates and times of induction to achieve process intensification.

## Materials and Methods

### Bacterial Strain and Vector Construction

#### Expression Strain

The glutathione reductase and thioredoxin reductase deficient (Δ*gor* Δ*trxB*) *E. coli* strain Rosetta gami B(DE3)pLysS was purchased from Merck, Darmstadt, Germany.

#### Expression Vector

The expression plasmid was created by Golden Gate (GG) cloning as previously described (Schreiber et al., 2017), with the addition of an 80x ELP (V48G16L16) with an N-terminal His_6_ tag at the fusion partner site, and a ΔI-CM intein at the cleavage site (Wood et al., 1999, 2000). A mutated version of the insect metalloproteinase inhibitor [IMPI (I38V)] was inserted at the product site (Hoffmann et al., 2019). As described by Schreiber et al. (2017), the 20-μL reaction mixture contained 40 fmol of each donor plasmid, 20 U T4 DNA ligase, 2 μL T4 DNA ligase buffer (Promega, Madison, WI, USA) and 10 U of BsaI (NEB, Ipswich, MA, USA). After warming to 37°C for 15 min, the GG mix was incubated for 50 cycles of 2 min at 37°C and 5 min at 16°C. Finally, the enzymes were heat-inactivated at 50°C for 15 min and 65°C for 5 min. The 2517-bp product (T7/lac+6xHis-ELP_80_-TrxB-intein-IMPI(I38V)-T7) was used for bacterial transformation (for plasmid see Supplementary Data).

#### Transformation, Plasmid Propagation, and Verification

Chemically competent *E. coli* NEB10-β cells (NEB) were transformed by adding 5 μL of the vector DNA to 80 μL of cells (optical density at 600 nm (Δ*OD*_600_) ≈ 13), in 100 mM CaCl_2_ + 15% glycerol (Carl Roth, Karlsruhe, Germany). The mixture was incubated on ice for 5 min followed by a 42°C heat shock for 60 s and then incubated on ice for another 5 min. We then added 500 μL lysogeny broth (LB) medium and allowed the cells to recover for 1 h at 37°C, shaking at 1,000 rpm on a thermomixer (Eppendorf, Hamburg, Germany). The cells were plated on LB agar containing 15 μg L^−1^ gentamycin (AppliChem, Darmstadt, Germany), and incubated at 37°C overnight. Individual colonies were picked and transferred to 5 mL LB medium containing antibiotics as above and incubated at 37°C, shaking at 250 rpm overnight. Cultures were harvested and plasmid DNA was purified using the NucleoSpin Plasmid EasyPure kit (Macherey-Nagel, Düren, Germany). Isolated DNA was digested and sequenced by Microsynth Seqlab (Seqlab Sequence Laboratories, Göttingen, Germany). Plasmids with correct inserts were introduced into chemically competent *E. coli* Rosetta-gami B(DE3)pLysS cells as described above, with all solid and liquid media supplemented with 34 μg L^−1^ chloramphenicol (Carl Roth).

### Media

Shaking flask and bioreactor media were prepared as recently described (Joachim et al., 2018) with a range of Fe(III)citrate concentrations. Each batch of medium was supplemented with 10 g L^−1^ glucose as the carbon source and 15 μg L^−1^ gentamycin, the latter to maintain selection pressure and ensure the growth of only those cells carrying the vector.

### Analytical Methods

#### Cell Growth

Absolute *OD*_600_ values were measured using a BioSpectrometer Kinetic (Eppendorf AG). At *OD*_600_ > 0.3, samples were diluted with 0.9% NaCl. The Δ*O*D_600_ was then calculated by subtracting the blank (*O*D_600, *b*_) from the sample absorbance (*O*D_600, *s*_) and multiplying by the dilution factor (F) as shown in Equation (1):

(1) $$\Delta OD_{600}\;=\;\left(OD_{600,\;s}\;-\;OD_{600,\;b}\right)\;\times \;F$$

The cell dry weight was determined using a MA100Q infrared moisture analyzer (Sartorius, Göttingen, Germany) or by weighing the dried pellet (48 h at 65°C) prepared from 2 mL of culture broth. The glucose concentration was determined using a Biosen C-line glucose/lactate analyzer (EKF Diagnostics, Cardiff, UK).

#### Target Protein Analysis

After cell disruption using BugBuster Mastermix (Merck), recombinant protein levels in the soluble fraction were determined with an enzyme-linked immunosorbent assay (ELISA) using THE plates coated with an antibody specific for the His_6_ tag (Genescript, Piscataway, NJ, USA). His_6_-tagged interleukin 6 (Firalis, Huningue, France) was used as a standard. A horseradish peroxidase (HRP)-coupled QIAexpress antibody specific for the His_6_ tag (Qiagen, Hilden, Germany) was used as a reporter. Standards, samples and the antibody were diluted in 0.2% bovine serum albumin (BSA; Carl Roth) in phosphate-buffered serine (PBS; Merck) and the signal was detected using the HRP substrate 1-Step Ultra TMB-ELISA (Thermo Fisher Scientific, Waltham, MA, USA). The plates were analyzed for 23 min at 1-min intervals at 370 nm in a Synergy HTX multi-mode reader running Gen5 software (BioTek Instruments, Winooski, VT, USA).

#### AMP Activity Assay

The activity of IMPI(I38V) was measured with a fluorescence-based assay using the metalloprotease thermolysin (Eisenhardt et al., 2015) and approximately 10 μg of ELP-coupled IMPI(I38V). Phosphoamidon (100 ng) was used as a positive control.

### Bioreactor Cultivations

Three different cultivation scales were compared in this study. Small-scale cultivations were carried out using a 0.5 L MiniBio 500 stirred-tank reactor system (Applikon Biotechnology, Delft, Netherlands) equipped with two Rushton impellers and a microsparger for aeration. Medium-scale cultivations were carried out using a 7.5 L Labfors3 bioreactor (Infors, Bottmingen, Switzerland) equipped with three Rushton impellers, three baffles and a standard sparger for aeration. Large-scale cultivation was carried out using a BIO BENCH 20 L bioreactor (Applikon Biotechnology) equipped with three Rushton impellers, four baffles and a standard sparger for aeration. All systems were also equipped with standard sensors for temperature, pH and oxygen saturation and BlueInOne Ferm offgas analyzers (BlueSens, Herten, Germany). The starting volume in the small-scale system was 0.25 L. Geometric similarity was ensured by applying Equation (2) (Junker, 2004), setting the vessel diameter of the small and large scale setups (*D*_1_ and *D*_2_) in relation to the corresponding liquid volumes (*V*_1_ and *V*_2_). This resulted in starting volumes of 2.86 L for the medium-scale experiments and 6.47 L for the large-scale experiments.

(2) $$\frac{D_{2}}{D_{1}}\;={\left(\frac{V_{2}}{V_{1}}\right)}^{\frac{1}{3}}$$

**Figure 1:**
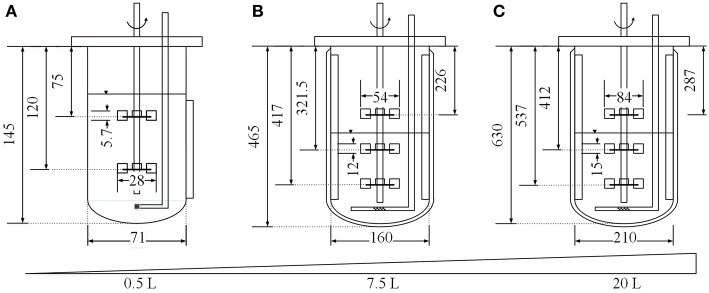
Dimensions (in mm) of the MiniBio 500 **(A)**, Labfors3 **(B)** and BIO BENCH 20 L **(C)** bioreactor systems.

The bioreactors were inoculated at an initial Δ*O*D_600_ of 0.1. After reaching a DO concentration of 30%, this value was maintained by a cascade of higher stirrer speeds, increasing aeration rates and finally the addition of oxygen. In small-scale experiments, the cascade consisted of increasing the stirrer speed from 1,000 to 1,600 rpm and the aeration rate from 0.1 to 1.0 vvm. The borders of the stirrer cascade in the medium-scale and large-scale experiments were determined by the criterion of an equal volumetric power input (*P/V*) (Equation 3).

(3) $$\frac{P_{G}}{V}\;=\frac{Ne_{G}\times \rho \times n^{3}\times d^{5}}{V}$$

where *n* [s^−1^] is the impeller speed, *d* [m] is the impeller diameter at the corresponding scale (Figure 1), ρ [kg m^−3^] is the density of the medium, and *Ne*_*G*_ is the gassed Newton number, calculated using Equation (4) (Henzler, 1982).

(4) $$Ne_{G}\;=z\times \frac{Ne_{0}+187\times Q\times Fr^{-0,32}\times {\left(\frac{d}{D}\right)}^{1,53}-4.6\times Q^{1,25}}{1+136\times Q\times {\left(\frac{d}{D}\right)}^{1,14}}$$

where *z* is the number of immersed impellers, *D* [m] is the diameter of the vessel (Figure 1) and *Ne*_0_≈ 4.9 (for *Fr* < 0.07(*D*/*d*)^3^; 0.2 < *d*/*D* < 0.42; *Re*>10^4^) (Henzler, 1982). Two dimensionless variables must be calculated, namely the Froude number (*Fr*) as shown in Equation (5)

(5) $$Fr\;=\frac{d\times n^{2}}{g}$$

and the gas flow number (*Q*) as shown in Equation (6)

(6) $$Q\;=\frac{F_{AIR}}{n\times d^{3}}$$

where *g* [m s^−1^] is the gravitational acceleration and *F*_*AIR*_ [m^3^ s^−1^] is the volumetric gas flow rate. By equating the *P/V* (Equation 3) of the larger scales to that of the small-scale system, the stirrer speed at the larger scale was calculated according to the stirrer speed in the small-scale experiments. The medium-scale stirrer cascade was therefore set to 754–1,207 rpm and that of the large scale was set to 465–744 rpm. The aeration cascade was set to 0.1–1 vvm for all scales. After the initial batch phase, a fed-batch phase was automatically initiated by the sharp increase in the DO signal after glucose depletion. The start feed rate was calculated using Equation (7).

(7) $$F(t_{0})=\frac{m_{X}\times (\frac{\mu_{set}}{Y_{\frac{X}{S}}}+m)}{c_{S,R}}$$

where *m*_*X*_ [g] is the current biomass in the vessel, *u*_*set*_ [h^−1^] is the growth rate, Y_*X*/*S*_ [g g^−1^] is the glucose consumption rate (0.35 g g^−1^), *m* is the maintenance-coefficient (0.04 g (g h)^−1^) and *c*_*S, R*_ is the glucose concentration in the feed reservoir (450 g L^−1^). The subsequent exponential feed rate was then calculated using Equation (8).

(8) $$F\left(t\right)=\;F\left(t_{0}\right)\times e^{\mu_{set}\times t}$$

### Determination of the Volumetric Mass Transfer Coefficient (*K_*L*_a*)

In the bioreactor systems, K_*L*_*a* values were determined by the direct online evaluation of exhaust gas analyzer data. By using a literature value for the molar volume *V*_*m*_ [m^3^ mol^−1^] and knowing the airstream $\overset{∙}{V_{Air}}$ [m^3^ h^−1^] and the volume of the liquid phase *V*_*L*_ [m^3^], as well as the mole fractions of oxygen *x*_*O*_2__ [-] and carbon dioxide *x*_*C*_*O*__2__ [-] in the ingas (α) and offgas (β) streams, the molar oxygen uptake rate (OUR) can be calculated as shown in Equation (9).

(9) $$OUR=\left(\frac{{\dot{V}}_{Air}}{V_{L}V_{m}}\right)\left(x_{O_{2}}^{\alpha }-x_{O_{2}}^{\beta }\frac{\left(1-x_{O_{2}}^{\alpha }-x_{CO_{2}}^{\alpha }\right)}{\left(1-x_{O_{2}}^{\beta }-x_{CO_{2}}^{\beta }\right)}\right)$$

By including the molar mass of oxygen *M*_*O*_2__ [g mol^−1^] and the calculation of $c_{L}^{*}$ [g m^−3^] (Schumpe et al., 1978), the K_*L*_*a* value can be calculated as an online variable (Equation 10):

(10) $$K_{L}a=OUR\times M_{O_{2}}\times \;\frac{1}{\left(c_{L}^{*}-c_{L}\right)}$$

### Optimization of Fe(III)Citrate Levels

The optimal Fe(III)citrate concentration (within the boundaries 0–2 g L^−1^) for IMPI expression was determined using a seven-level factorial design at concentrations of 0, 0.33, 0.67, 1, 1.33, 1.67, and 2 g L^−1^. The cultures were inoculated at Δ*O*D_600_ = 0.1 and incubated at 37°C in 500 mL baffled shake flasks containing 50 mL medium, shaking at 250 rpm on a Multitron Standard shaker (Infors). At Δ*O*D_600_ = 1, IMPI expression was induced by addinig isopropyl-β-d-thiogalactopyranoside (IPTG; Carl Roth) at a final concentration of 1 mM followed by incubation for 4 h as above. The cells were then harvested by centrifugation (5,236 × g for 10 min at 4°C) in a Sigma 6-16 KS centrifuge equipped with a 11,650 rotor and four 13,650 cups (Sigma, Osterode am Harz, Germany). The pellet was disrupted for IMPI activity testing as described above. The results were evaluated using Design Expert v11 (Stat Ease, Minneapolis, MN, USA).

### Optimization of Induction

Small-scale experiments to determine the best induction conditions in MiniBio 500 reactors were conducted using 250 mL of the chemically defined minimal medium supplemented with 7 g L^−1^ glucose at 37°C. The pH was maintained at 6.8 by adding 25% aqueous ammonia, which also provided nitrogen. DO levels were maintained at ≥30% by a cascade in which the stirrer speed was gradually increased from 1,000 to 1,600 rpm, then the air flow rate was increased from 0.1 to 1.67 vvm, and finally the O_2_ flow was increased from 0 to 0.47 vvm (relative to the initial volume). The fed-batch phase started with an initial feeding rate of *F*(t_0_) = 4.428 ×10^−4^ L h^−1^ after a sudden rise in the DO levels due to the depletion of glucose. The growth rate of the exponential feeding function was set to μ = 0.1 h^−1^. When the culture reached Δ*O*D_600_ = 17, induction conditions were investigated using response surface methodology and a central composite design. The design consisted of a total of 36 runs, investigating inductor (IPTG) levels in the range 0.1–2.1 mM, induction durations of 0.63–7.36 h and induction temperatures of 31.3–44.7°C. At given time points, samples were taken to measure Δ*OD*_600_, cell dry weight and product titers as described above.

### Evaluation of Scale-Up

To determine the success of scale-up in terms of Δ*O*D_600_, K_*L*_*a* and product titers, the data for each scale were plotted as parity plots against the other scales followed by linear fitting. *R*^2^ was used to evaluate the linear dependency, and the difference in the resulting slope of the fit (s_*F*_) to the slope of the parity line (s_*P*_) was used to quantify the success of scale-up (Equation 11).

(11) $$\Delta s=\left|s_{P}-s_{F}\right|\times 100\%$$

## Results and Discussion

### Optimization of Fe(III)Citrate Concentration

**Figure 2:**
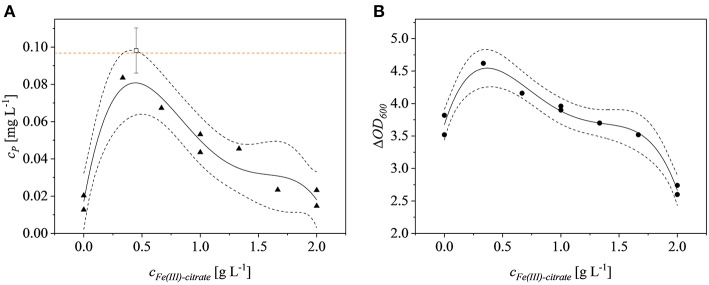
Fe(III)-citrate optimization. **(A)** Product concentration of the model runs (▴) and of the confirmation runs (□). The orange dotted line represents the product concentration achieved in LB complex medium with no additional Fe(III)-citrate. **(B)** Δ*O*D_600_ (•) at the time of harvest is dependent on the Fe(III)-citrate concentration. Dotted black lines represent the confidence bands (95%) of the plotted model (solid line).

Iron is a critical factor for the growth of *E. coli* Rosetta gami B(DE3)pLysS cells (Joachim et al., 2018) but the impact of different iron concentrations on recombinant protein expression has not been tested in detail. We found that the effect of iron was significant (*p* = 0.0322). The quartic model based on the experimental data had a *p*-value of 0.0038 (*F* = 17.48) and had no significant lack of fit (*p* = 0.1839). The model was fitted with an adjusted *R*^2^ value of 0.8799. Optimization for the highest product titers revealed that the optimal Fe(III)citrate-hydrate concentration was 0.44 g L^−1^, which was selected for the iron-enriched minimal medium hereafter termed M9i. The effect of Fe(III)citrate-hydrate on the Δ*O*D_600_ at the time of harvest was also significant (*p* = 0.0051) as was the corresponding quartic model (*p* = 0.0004), which had no significant lack of fit (*p* = 0.5412) resulting in an adjusted *R*^2^ value of 0.9543. The model was confirmed by testing biological triplicates at 0.44 g L^−1^ Fe(III)citrate-hydrate, resulting in fusion protein yields of 0.098 ± 0.01 mg L^−1^. The 95% confidence interval of the model was met with these findings and the product concentration was similar to the 0.097 mg L^−1^ achieved using LB complex medium (Figure 2). The optimal Fe(III)citrate-hydrate concentration for recombinant protein expression in this strain (0.44 g L^−1^) agreed well with the optimum of 0.49 g L^−1^ for non-induced growth (Joachim et al., 2018). A possible explanation for the decrease of the product titers at concentrations above the determined optimum might be the negative effect of formed radicals on the growth of *E. coli* at high iron concentrations (Gelvan, 1997; Chamnongpol et al., 2002). Although the product titer of 0.098 ± 0.01 mg L^−1^ using M9i medium was similar to the low levels achieved using complex LB medium, such product titers are not uncommon for the production of AMPs in *E. coli*. For example, the yield of CM4 coupled to a thioredoxin tag was 1.4 mg L^−1^ (Zhou et al., 2009), and this declined to 0.6 mg L^−1^ when the same product was coupled to an ELP-intein tag (Shen et al., 2010). Although (Gaciarz et al., 2017) found no evidence for the efficient production of soluble proteins in redox-engineered *E. coli* strains growing in chemically defined media, we have shown that our M9i medium achieves comparable product titers to the same *E. coli* strains growing on complex LB medium.

### Optimization of Induction Parameters

We found that the induction parameters had a significant influence on the yield of recombinant protein based on a cubic model (*p* < 0.0001) with no significant lack of fit (*p* = 0.4740). The fit of the model was possible with a predicted *R*^2^ value of 0.7335. Equation (12) shows the corresponding third-order polynomial equation, giving the recombinant protein concentration c_*P*_ [mg L^−1^] as a function of the concentration of inducer c_*IPTG*_ [mM], the duration of induction t_*ind*_ [h] and the temperature *T* [°C]:

(12) $$\begin{array}{l} \sqrt{c_{P}}=52.14653+1.15242\;c_{IPTG}+0.025100\;t_{ind}-4.54991\;T\\ \;-0.504829\;c_{IPTG}{}^{2}+0.130092\;T^{2}-0.001220\;T^{3} \end{array}$$

**Figure 3:**
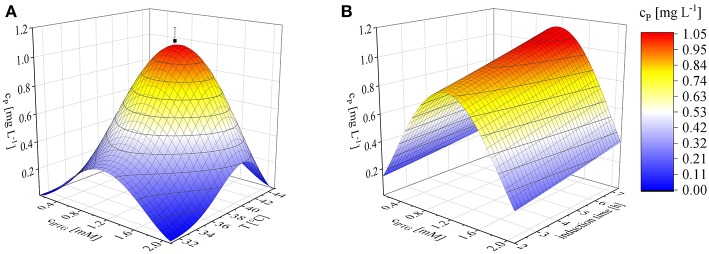
Optimization of induction to maximize the recombinant protein concentration (c_*P*_) using Equation (12), shown as 3D plots. **(A)** Concentration of inducer against temperature after induction for 6 h. **(B)** IPTG concentration against the duration of induction at a constant temperature of 40°C. Confirmation runs (■) under the optimal conditions predicted by the model were performed using biological triplicates.

Numerical optimization revealed an optimum, within the investigated parameter ranges, of 1.1 mM IPTG, an induction temperature of 40°C and at the longest induction time we evaluated (7.36 h). Given the model (Equation 12), the projected maximum product concentration c_*P*; *max*_ was 1.1 mg L^−1^. The mean of three confirmation runs, under the predicted optimal conditions, was 1.03 ± 0.06 mg L^−1^ and thereby fitted the 95% confidence interval of the model and confirmed its validity (Figure 3). Some earlier studies found similar optimal induction conditions, such as 1 mM IPTG and no difference within the temperature range 37–42°C (Collins et al., 2013). Others achieved the highest recombinant protein yields at 0.1 mM IPTG and 22°C (Wang et al., 2005). The optimization of induction is therefore still a matter of trial and error (Papaneophytou and Kontopidis, 2014). The induction optimization experiments were performed at an exponential feed rate with a growth rate of 0.15 h^−1^ and at an induction Δ*OD*_600_ of 17 in the MiniBio 500 bioreactor. To determine the influence of higher feed rates and higher cell concentrations at the time of induction, an appropriate scale-up strategy was required.

### Scaling Up the Production of IMPI

**Figure 4:**
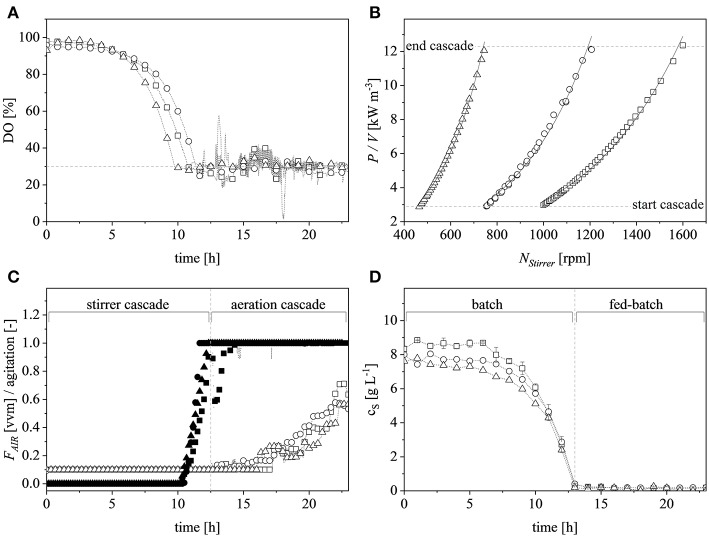
Scaling up by applying the combined criteria of constant dissolved oxygen (DO) levels and matching volumetric power input (*P/V*) at the limits of the stirrer cascade at the 0.5 L (□), 7.5 L (°) and 20 L (Δ) scales. **(A)** DO trends at the different scales during the course of cultivation. The dashed gray line represents the scale-up criteria of 30% DO. **(B)** *P/V* plotted against the agitation of the stirrer at the corresponding scale. The dashed gray lines show the cascade limits whereas the solid black lines show the exponential fits (*R*^2^ > 0.99 for all scales). **(C)** Normalized stirrer agitation over the course of a representative cultivation at the 0.5 L (■), 7.5 L (•), and 20 L (▴) scales, and after reaching the end of the cascade (dashed gray line) the subsequent aeration cascade over the course of a representative cultivation at the 0.5 L (□), 7.5 L (°), and 20 L (Δ) scales. **(D)** Glucose concentration over the course of a representative cultivation in the supernatant of the 0.5 L (□), 7.5 L (°), and 20 L (Δ) scales. Gray, dashed, vertical line depicts the total glucose depletion and therefore the start of the exponential feed profile.

A vessel with a higher working volume was needed to increase the feed rate and hence the growth rate, allowing us to investigate the influence of induction at higher cell densities. To make sure the observed changes could be attributed to changes in the growth rate and cell density rather than the scale *per se*, the selected parameters (μ_*set*_ = 0.15 h^−1^ and induction at Δ*OD*_600_ = 17) were scaled up in parallel. The scale-up criteria applied in biotechnological processes have been widely reviewed (Junker, 2004; Schmidt, 2005; Garcia-Ochoa and Gomez, 2009). Junker (2004) emphasized constant DO levels as an appropriate scale-up criterion for microbial bioprocesses, as long as the DO is kept above 30–70% saturation (DO-const.). Controlling the DO levels solely by increasing the stirrer speed can lead to undesirable morphological changes (Okada and Iwamatu, 1997). Therefore, a combined scale-up strategy of a constant DO level (30% saturation) and matching *P/V* at the borders of the stirrer cascade was applied in this study. All cultivations were started at a *P/V* of ~3 kW m^−3^. Having reached the scale-up criteria (Figure 4A), a stirrer cascade was used to increase the *P/V* to a final value of ~12 kW m^−3^. During this phase, the influence of the stirrer speed (N_*Stirrer*_) on *P/V* followed Equation (13) and the corresponding parameters are shown in Table 1. The steepening slope showing the progressively higher influence of the stirrer speed on *P/V* as the scale increased from 0.5 to 7.5 and finally 20 L was not surprising because the impeller diameter in Equation (3) is raised to the power 5, and thus has a massive impact on the *P/V* when the stirrer speed is increased (Figure 4B).

(13) $$\frac{P}{V}=a\times e^{b\;\times \;N_{Stirrer}}$$

**Table 1:** Fitting parameters of the three scales (Equation 13).

**Scale**	**a**	**b**	***R*^**2**^**
0.5 L	0.31	0.0023	0.9956
7.5 L	0.31	0.0031	0.9932
20 L	0.31	0.0050	0.9963

**Figure 5:**
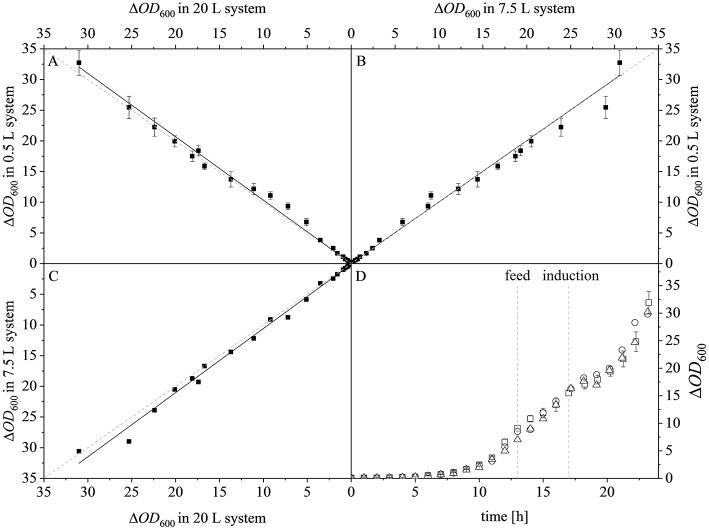
Optical densities (Δ*OD*_600_) at different scales. **(A)** Parity plot of Δ*OD*_600_ at the 20 L (x-axis) and 0.5 L (y-axis) scales, linear fit (*y* = *0.019* + *1.032x*), *R*^2^ = 0.9940. **(B)** Parity plot of Δ*OD*_600_ at the 7.5 L (x-axis) and 0.5 L (y-axis) scales, linear fit (*y* = *0.033* + *1.001x*), *R*^2^ = 0.9943. **(C)** Parity plot of Δ*OD*_600_ at the 20 L (x-axis) and 7.5 L (y-axis) scales, linear fit (*y* = *0.116* + *1.046x*), *R*^2^ = 0.9969. Solid black lines show the linear fits and the dashed gray lines represent lines of parity. **(D)** Δ*OD*_600_ of the 0.5 L (□), 7.5 L (°), and 20 L (Δ) scales during the course of each fermentation.

**Figure 6:**
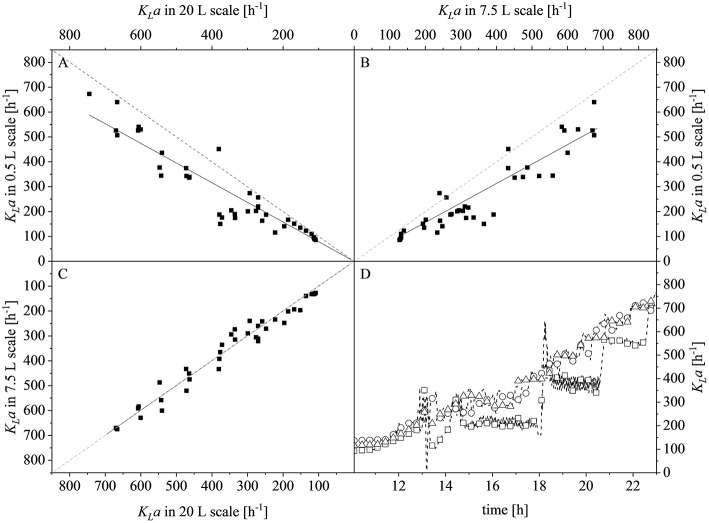
Comparison of volumetric mass transfer coefficients (K_*L*_*a*) at different scales and during the course of cultivation. **(A)** Parity plot of K_*L*_*a* for the 20 L (x-axis) and 0.5 L (y-axis) scales with a slope of 0.7903, *R*^2^ = 0.9595. **(B)** Parity plot of K_*L*_*a* for the 7.5 L (x-axis) and 0.5 L (y-axis) scales with a slope of 0.7820, *R*^2^ = 0.9634. **(C)** Parity plot of K_*L*_*a* for the 20 L (x-axis) and 7.5 L (y-axis) scales with a slope of 0.9984, *R*^2^ = 0.9895. Solid black lines show the linear fits and dashed gray lines represent lines of parity. **(D)** K_*L*_*a* values determined online for the 0.5 L (□), 7.5 L (°), and 20 L (Δ) scales during the course of each fermentation. Data points are connected by dashed lines to guide the eyes.

After reaching the end of the stirrer cascade, the DO level was maintained at 30% by increasing the aeration rate from 0.1 to 1 vvm relative to the starting volume (Figure 4C). Following the initial batch phase, the fed-batch began after complete glucose depletion with the appropriate feed rate for each scale. The μ_*set*_ of 0.15 h^−1^ led to a glucose-limited fed-batch profile for all scales (Figure 4D). The growth rate in the initial batch phase was 0.43 h^−1^ for the 0.5 and 7.5 L reactors and 0.42 h^−1^ (corresponding to a generation time of approximately 1.6 h) at the 20 L scale (Figures 5A–C). After initiating the feeding, inducing with 1.1 mM IPTG and raising the temperature to 40°C, the growth rate fell to 0.11, 0.12, and 0.11 h^−1^ for the 0.5, 7.5, and 20 L scales, respectively (Figure 5D). Plotting the Δ*OD*_600_ values of the 0.5 and 7.5 L cultivations against each other revealed a linear dependency, with a difference to the line of parity of Δ*s* = 0.1% (Figure 5B). A comparison of the Δ*OD*_600_ values of the 7.5 and 20 L scales also revealed a linear dependency and a Δ*s* value of 4.6% (Figure 5C). Finally, the direct scale-up from 0.5 to 20 L revealed a linear dependency with a Δ*s* value of 3.2% (Figure 5A). For all linear fits, *R*^2^ > 0.99. The Δ*OD*_600_ slopes for each comparison revealed <5% deviation from the line of parity at the different scales when the scale-up strategy of DO-const. and a matching *P/V* at the borders of the stirrer cascade was applied. Islam et al. (2008) showed that different K_*L*_*a* values severely affected the success of scaling up processes based on *E. coli*. Therefore, a scale-up methodology which duplicates the K_*L*_*a* trend during the course of cultivation should improve process reproducibility when changing scales. The DO reached 30% ~10 h after inoculation and was subsequently kept constant (Figure 4C). From this time point, it was possible to calculate the volumetric mass transfer coefficient online. The exponential growth in the K_*L*_*a* value during cultivation came as no surprise, given earlier reports of similar trends (Castan and Enfors, 2000). Moreover, the growth rates of the exponential functions (*b* in Equation 14) showed reasonable agreement with the μ_*set*_ of 0.15 h^−1^ (Table 2). As discussed above for the Δ*OD*_600_ values, plotting the K_*L*_*a* values of the smaller and larger scales against each other revealed a linear dependency (Figure 6). When the K_*L*_*a* of the 0.5 L system was compared to the larger scales, the difference to the line of parity (Δ*s*) was 20.97% for the 7.5 L system and 21.8% for the 20 L system. However, when the two larger systems were compared the Δ*s* value was only 0.16%. The discrepancy between the 0.5 L system and the larger scales was attributed to a technical artifact in the aeration strategy. A more or less pronounced stepwise increase in the air flow rate was observed at the different scales (Figure 4C) and had a more severe impact in the small-scale process. This stepwise increase in aeration was also found in the K_*L*_*a* values, hence the difference in the volumetric mass transfer coefficient from the 0.5 L scale to the larger scales.

**Table 2:** Fitting parameters of the three scales (Equation 14).

**Scale**	**a**	**b**	***R*^**2**^**
0.5 L	20.80	0.1481	0.8642
7.5 L	37.99	0.1294	0.9689
20 L	36.11	0.1317	0.9648

(14) $$K_{L}a=a\times e^{b\;\times t}$$

**Figure 7:**
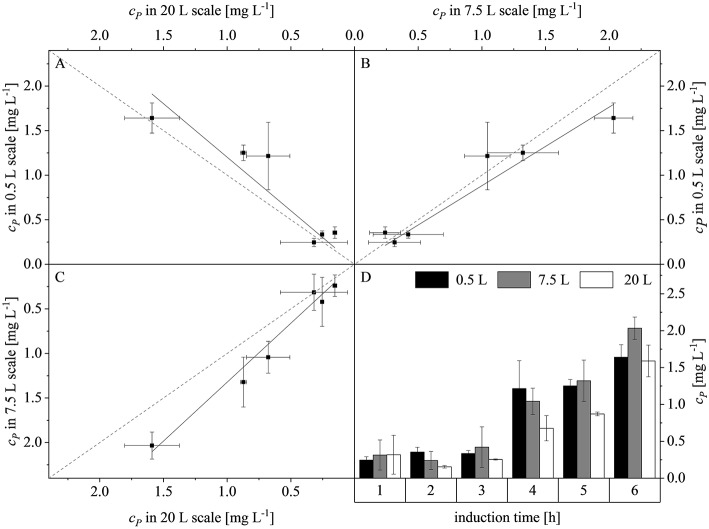
Concentration of recombinant protein (c_*P*_) compared among the production scales and during the course of induction. **(A)** Parity plot of c_*P*_ at the 20 and 0.5 L scales (adj. *R*^2^ = 0.93, with a slope of 1.20). **(B)** Parity plot of c_*P*_ at the 7.5 and 0.5 L scales (adj. *R*^2^ = 0.98, with a slope of 0.88). **(C)** Parity plot of c_*P*_ at the 7.5 and 20 L scales (adj. *R*^2^ = 0.99, with a slope of 1.32). Solid black lines show the linear fits and dashed gray lines represent lines of parity. **(D)** Trend of c_*P*_ during induction at the 0.5, 7.5, and 20 L scales.

Plotting the product concentrations (c_*P*_) of the 0.5 and 7.5 L scales against each other revealed a linear dependency with a slope of 0.88. Similarly there was a linear dependency between the 0.5 and 20 L scales with a slope of 1.20, and between the 7.5 and 20 L scales with a slope of 1.32. Hence, the difference between the slopes and the line of parity (Δ*s*) at the three scales was 12 ± 4.2, 20 ± 5.9, and 32 ± 8.5%, respectively (Figures 7A–C). The high deviations were attributed to the intrinsic high error rate of the immunoassay. Weber et al. (2013) proposed that an inter-assay coefficient of variance of 22% is acceptable for ELISAs using biological samples. The highest product concentrations were reached after 6 h at all three scales: 0.5 L (1.64 ± 0.16 mg L^−1^), 7.5 L (2.03 ± 0.23 mg L^−1^), and 20 L (1.59 ± 0.21 mg L^−1^). There were no significant differences among the scales (*p*-values of 0.0810, 0.8531, and 0.0940, respectively). The scale-up of the fed-batch process was successful in terms of growth, volumetric mass coefficient and protein production. The similar behavior of the cultures when scale transfer was implemented using the combined scale-up criteria of a constant DO concentration and matching *P/V* at the borders of the stirrer cascade allowed us to test changes in the feed rate and time of induction at the 7.5 L scale, and also at the 20 L scale.

### Investigation of the Feed Rate and Time of Induction

**Figure 8:**
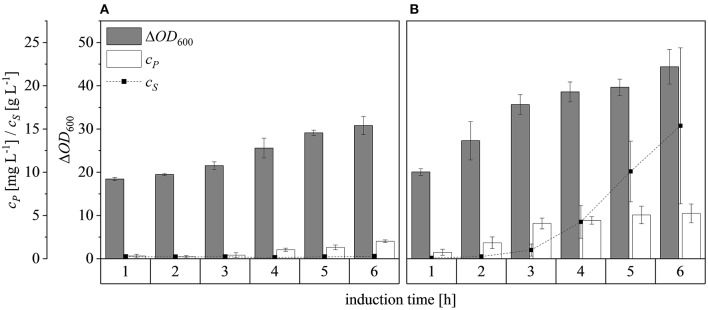
Optical densities (Δ*OD*_600_), product concentrations (c_*P*_) and substrate concentrations (c_*S*_) in the supernatant during induction at the 7.5 L scale. **(A)** Feed growth rate of 0.15 h^−1^ **(B)** Feed growth rate of 0.25 h^−1^. Hatched lines are provided to improve clarity.

**Figure 9:**
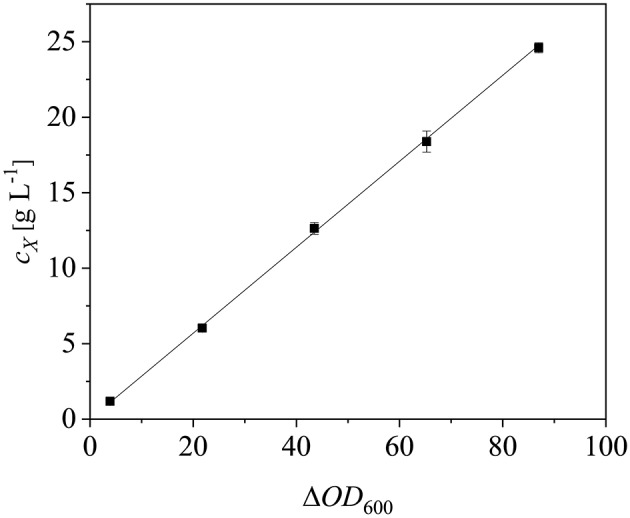
Correlation between cell dry weight (c_*X*_) and optical density (Δ*OD*_600_). Error bars show standard deviations of triplicates. The data were fitted linearly (*R*^2^ = 0.9994).

After scaling up the established process from 0.5 to 7.5 L, we investigated the effect of increasing the feed rate. The specific growth rate of *E. coli* is known to affect plasmid stability and recombinat protein production in fed-batch processes (Hellmuth et al., 1994; Ramalingam et al., 2007). A feed rate (μ_*set*_) of ~60% μ_*max*_ should not result in the accumulation of additional acetate (Vidal et al., 2005). Others have reported that growth rates below 0.35 h^−1^ in minimal medium inhibit acetic acid formation (Choi et al., 2006). Increasing the μ_*set*_ from 0.15 to 0.25 h^−1^ (~60% of the μ_batch_) increased the maximum Δ*OD*_600_ from 30.8 ± 2.1 to 44.4 ± 4.0 (Figure 8). The concentration of recombinant protein improved by ~2.7 fold from 2.03 ± 0.23 mg L^−1^ to 5.25 ± 1.08 mg L^−1^. These findings are in line with earlier reports of improved product titers at higher growth rates (Sandén et al., 2003). Although the peptide chain elongation rate stays constant, the number of ribosomes per cell increases with increasing growth rate, which may explain the enhanced productivity at higher feed rates (Maaløe, 1979; Farewell and Neidhardt, 1998). In some reports, product concentrations increase with the growth rate up to a certain point and then decline (Siurkus et al., 2010). Although no glucose accumulated in the process with a μ_*set*_ of 0.15 h^−1^, glucose started to accumulate ~3 h after induction when μ_*set*_ was increased to 0.25 h^−1^ at Δ*OD*_600_ = 17. The premature termination of growth and production may reflect the use of a discontinuous pump in the Labfors3 bioreactor. Enhancing the feed rate in this system leads to higher oscillations in glucose availability and subsequently in DO levels. Such oscillating substrate availability (described as “feast-then-famine”) can promote the accumulation of acetate (Neubauer et al., 1995; Eiteman and Altman, 2006). The production of acetate is a loss in net carbon and therefore economically not desirable (Jensen and Carlsen, 1990). Even more so, acetate accumulation is inhibitory to *E. coli* growth (Luli and Strohl, 1990; Hahm et al., 1994) and recombinant protein production (Jensen and Carlsen, 1990; Koh et al., 1992). Earlier we hypothesized that *E. coli* Rosetta gami B(DE3)pLysS cells may contain higher levels of intracellular NAD(P)H (Joachim et al., 2018). A higher NADPH:NAD ratio can occur when approaching the threshold growth rate (Vemuri et al., 2006). A combination of these factors may explain the sensitivity of Rosetta gami B(DE3)pLysS cells to glucose fluctuation. After plotting the Δ*OD*_600_ against the cell dry weight (c_*X*_) of a dilution series and linear fitting with an adjusted R^2^ value of 0.9994, the c_*X*_[g L^−1^] was calculated using Equation (15), with a standard error of the slope of 0.00297 g L^−1^. Product yields (Y_*P*/*X*_) [mg g^−1^] improved from 0.23 ± 0.001 mg g^−1^ to 0.42 ± 0.04 mg g^−1^ by increasing the feed growth rate from 0.15 h^−1^ to 0.25 h^−1^ (biomass calculated using correlation from Figure 9).

(15) $$c_{X}=0.2747\;\frac{g}{L}\times \Delta OD_{600}$$

**Figure 10:**
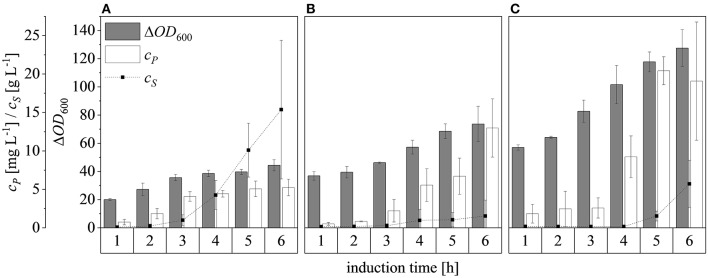
Optical densities (Δ*OD*_600_), product concentrations (c_*P*_) and substrate concentrations (c_*S*_) in the supernatant after induction at Δ*OD*_600_ values of 17 **(A)**, 30 **(B)**, and 50 **(C)**. Experiments were performed at the 7.5 L scale with a μ_*set*_ of 0.25 h^−1^ after induction. Hatched lines are provided to improve clarity.

Changing the Δ*OD*_600_ at induction from 17 to 30 and then 50, and subsequently feeding at a rate of μ_*set*_ = 0.25 h^−1^, resulted in final Δ*OD*_600_ values after induction for 6 h of 44.4 ± 4.0, 73.7 ± 12.5, and 127.5 ± 13.1, respectively (Figure 10). Although glucose accumulated 3 h after induction at Δ*OD*_600_ = 17, only a small amount accumulated after induction at Δ*OD*_600_ = 30 and it started to accumulate ~5 h after induction at Δ*OD*_600_ = 50. For all cases, both growth and protein production stagnated once glucose accumulation was detected. Possible reasons for the sudden growth and production stagnation might be the inhibitory effect of formed by-products (Jensen and Carlsen, 1990) or the metabolic overload due to the IPTG induction (Cserjan-Puschmann et al., 1999). An abrupt growth stagnation, paired with stagnating recombinant protein production and a sudden rise in glucose levels, in induced glucose limited fed-batch operations, was also reported by Pinsach et al. (2008). An overload of the host cell due to the induction pulse was emphasized as the reason behind the systems collapse. The highest product titers were 5.3 ± 1.1 mg L^−1^, 13.0 ± 3.8 mg L^−1^, and 20.4 ± 1.8 mg L^−1^ at induction Δ*OD*_600_ values of 17, 30, and 50, respectively. The ability of high cell densities to significantly increase recombinant protein production in *E. coli* has been reported before (Choi et al., 2006; Sivashanmugam et al., 2009). In agreement with previous reports (Yee and Blanch, 1992; Galloway et al., 2003), we found that the point of induction showed a profound effect on the product yields. When the induction Δ*OD*_600_ was changed from 17 to 30, the product yield increased from 0.42 ± 0.04 to 0.72 ± 0.05 mg g^−1^, but it decreased to 0.61 ± 0.01 mg g^−1^ when the culture was induced at Δ*OD*_600_ = 50 (Figure 7D). The volumetric production yield was still highest when induced at Δ*OD*_600_ = 50, due to the greater quantity of biomass in the vessel.

### AMP Activity

**Figure 11:**
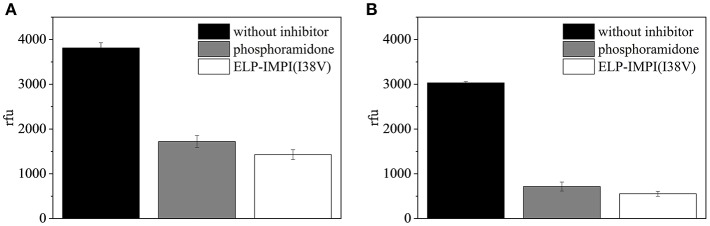
Fluorescence-based assay to test the activity of IMPI against thermolysin. A replicate assay lacking IMPI served as a negative control, and phosphoramidon was included as a positive control. Error bars represent the standard deviation of triplicates. **(A)** ELP-IMPI(I38V) produced using LB complex medium, induction at 37°C with 1.0 mM IPTG. **(B)** ELP-IMPI(I38V) produced using M9i minimal medium, induction at 40°C with 1.1 mM IPTG.

The ELP-coupled IMPI(I38V) samples produced using the standard process (LB complex medium, induction at 37°C with 1.0 mM IPTG) and under the optimized conditions (M9i minimal medium, induction at 40°C with 1.1 mM IPTG) were each found to be active (Figure 11). Even though protein folding is known to be enhanced by reducing the induction temperature (Georgiou and Valax, 1996), we found that a higher temperature was beneficial for IMPI. This is supported by earlier reports in which a higher induction temperature did not result in a significant increase in the quantity of recombinant protein segregating into inclusion bodies (Larentis et al., 2011). Furthermore, higher temperatures can also improve the synthesis of some proteins in *E. coli* (Yamamori et al., 1978) potentially explaining the positive effect we observed when the temperature was increased at the time of induction.

## Conclusion

The minimal medium enriched with Fe(III)-citrate (M9i) was comparable in performance to LB complex medium when recombinant ELP-coupled IMPI was produced in the redox-engineered *E. coli* strain Rosetta gami B(DE3)pLysS. Optimal induction was achieved at 40°C with 1.1 mM IPTG. Suitable scale-up criteria for the multi-cascaded fed-batch process were found by combining a constant DO and matching volumetric power inputs at the borders of the stirrer cascade. Changes in the exponential feed rate and the time of induction improved the titers of the recombinant protein. The time of harvest was determined by growth depletion and subsequently glucose accumulation. In summary, process intensification resulted in a scalable bioprocess with enhanced productivity, allowing us to develop a simple and controllable process for the production of an active AMP in *E. coli*. The use of self-aggregating ELP tags simplifies the process by allowing the integration of upstream production and downstream processing. Furthermore, the proposed sensitivity of the *E. coli* strain to fluctuations in feed concentration and oxygen saturation is striking and should be investigated in more detail.

## Data Availability

All datasets generated for this study are included in the manuscript.

## Author Contributions

MJ designed and performed the experiments and wrote the paper. NM and JS assisted with the experiments. DG and PC helped to draft and revise the manuscript, and supervised the research. All authors contributed to manuscript revision, read, and approved the submitted version.

### Conflict of Interest Statement

The authors declare that the research was conducted in the absence of any commercial or financial relationships that could be construed as a potential conflict of interest.
