# Supplementary material for: Process Intensification for an Insect Antimicrobial Peptide Elastin-Like Polypeptide Fusion Produced in Redox-Engineered Escherichia coli
Source: Front Bioeng Biotechnol. 2019 Jun 27;7:150. doi: 10.3389/fbioe.2019.00150 (PMC6610315; doi:10.3389/fbioe.2019.00150)
Supplement: Supplementary file 1 [file Data_Sheet_1.pdf]

# *Supplementary Material*

# Process intensification for an insect antimicrobial peptide elastin-like polypeptide fusion produced in redox-engineered *Escherichia coli*

**Mathias Joachim[1,2], Nicolas Maguire[3], Johannes Schäfer[1], Doreen Gerlach[3], Peter Czermak[1,2,3,*]**

[1]Institute of Bioprocess Engineering and Pharmaceutical Technology, University of Applied Sciences Mittelhessen, Giessen, Germany

[2]Faculty of Biology and Chemistry, Justus Liebig University, Giessen, Germany

[3]Department of Bioresources of Fraunhofer Institute for Molecular Biology and Applied Ecology IME, Giessen, Germany

*** Correspondence:**
Prof. Dr.-Ing. Peter Czermak
peter.czermak@lse.thm.de

## 1 Supplementary Data

The nucleic acid sequence of the The 2517-bp product (T7/lac+6xHis-$ELP_{80}$–TrxB-intein-IMPI(I38V)-T7) used for bacterial transformation:

taatacgactcactataggggaattgtgagcggataacaattcccctctagaaataattttGTTTAACTTTAAGAAGGAGATAT
ACATatgcatcaccatcaccatcacAGGATGGTGGGAGTTCCAGGAGTCGGAGTTCCAGGTGGAGGA
GTACCTGGTTTGGGTGTTCCTGGTGTAGGAGTCCCTGGTGTAGGTGTTCCTGGTGTCGGA
GTACCAGGTGGAGGAGTCCCTGGATTGGGTGTTCCTGGTGTTGGTGTGCCAGGTGTGGG
AGTTCCAGGTGTCGGTGTACCTGGTGGAGGTGTTCCTGGTTTGGGTGTACCTGGTGTCGG
TGTACCTGGTGTAGGAGTACCAGGTGTCGGAGTACCTGGAGGAGGTGTCCCTGGTTTGG
GTGTTCCTGGAGTTGGAGTGCCAGGTGTGGGAGTTCCAGGTGTCGGAGTTCCTGGAGGT
GGTGTCCCTGGATTGGGAGTTCCTGGTGTTGGAGTACCTGGAGTCGGTGTTCCCGGTGTT
GGTGTTCCTGGTGGAGGTGTTCCCGGATTGGGAGTCCCTGGAGTCGGAGTGCCAGGTGT
GGGAGTTCCAGGTGTGGGTGTTCCTGGTGGTGGTGTTCCTGGACTGGGAGTACCTGGTGT
TGGTGTACCAGGTGTCGGTGTTCCTGGCGTTGGAGTGCCTGGAGGAGGTGTTCCTGGATT
GGGAGTACCCGGTGTTGGTGTGCCAGGTGTGGGAGTTCCAGGAGTCGGAGTTCCAGGTG
GAGGAGTACCTGGTTTGGGTGTTCCTGGTGTAGGAGTCCCTGGTGTAGGTGTTCCTGGTG
TCGGAGTACCAGGTGGAGGAGTCCCTGGATTGGGTGTTCCTGGTGTTGGTGTGCCAGGT
GTGGGAGTTCCAGGTGTCGGTGTACCTGGTGGAGGTGTTCCTGGTTTGGGTGTACCTGGT
GTCGGTGTACCTGGTGTAGGAGTACCAGGTGTCGGAGTACCTGGAGGAGGTGTCCCTGG
TTTGGGTGTTCCTGGAGTTGGAGTGCCAGGTGTGGGAGTTCCAGGTGTCGGAGTTCCTGG
AGGTGGTGTCCCTGGATTGGGAGTTCCTGGTGTTGGAGTACCTGGAGTCGGTGTTCCCGG
TGTTGGTGTTCCTGGTGGAGGTGTTCCCGGATTGGGAGTCCCTGGAGTCGGAGTGCCAG
GTGTGGGAGTTCCAGGTGTGGGTGTTCCTGGTGGTGGTGTTCCTGGACTGGGAGTACCTG
GTGTTGGTGTACCAGGTGTCGGTGTTCCTGGCGTTGGAGTGCCTGGAGGAGGTGTTCCTG

GATTGGGAGTACCCGGTGTTGGTGTGCCAGGTGGGCCTagtgaaagcgacaaaattatccacctgacggacg
acagtttcgacacggatgttctgaaagccgatggcgcgattctggttgacttctgggcggaatggtgcggcccgtgtaaaatgattgcaccgatcct
ggatgaaattgctgacgaatatcagggcaaactgaccgtggcgaaactgaacatcgatcaaaatccgggtaccgccccgaaatacggcattcgt
ggtatcccgacgctgctgctgtttaaaaacggtgaagttgcggccaccaaagtcggtgccctgtcaaaaggtcaactgaaagaattcctggacgc
aaatctggcgGAGCAAGGAAGTGGAAGTGCATTGGCAGAGGGAACAAGAATTTTTGACCCAG
TCACCGGAACTACTCATAGAATCGAAGATGTCGTTGGAGGAAGAAAGCCAATTCATGTT
GTCGCTGCCGCAAAAGATGGAACATTGCACGCTAGACCTGTTGTCTCATGGTTTGATCA
AGGAACTAGAGACGTTATTGGTCTTAGAATCGCAGGTGGAGCTATCTTGTGGGCTACCC
CAGATCATAAGGTCTTGACTGAATACGGTTGGAGAGCTGCCGGAGAGTTGAGAAAAGGT
GACAGAGTTGCTCAACCTAGAAGATTTGATGGTTTCGGAGACTCTGCCCCAATTCCTGCA
AGAGTTCAGGCATTGGCTGATGCCCTTGATGACAAGTTTTTGCACGACATGCTTGCTGAA
GAGTTGAGATATTCCGTCATCAGAGAAGTTCTTCCAACTAGAAGAGCTAGAACATTCGG
ATTGGAAGTCGAAGAGTTGCATACTCTTGTTGCCGAGGGTGTTGTCGTTCACAACggaagtat
tgtgctgatttgtaacggtggtcacgaatactacgaatgtggcggtgcttgcgataatgtctgtgctgatctgcatatccaaaacaaaaccaactgcc
cgatcatcaacgtgcgttgtaacgataaatgctactgtgaagatggctacgcgcgcgacgtgaatggtaaatgcattccgatcaaagactgtccga
aaatccgtagctaatgggtaattgattaatacctaggctgctaaacaaagcccgaaaggaagctgagttggctgctgccaccgctgagcaataact
agcataaccccttggggcctctaaacgggtcttgaggggttttttg
